# Transcranial ultrasound combined with intravenous metformin-loaded oxygenated microbubbles attenuates noise-induced hearing loss in mice

**DOI:** 10.1080/10717544.2025.2576220

**Published:** 2025-11-05

**Authors:** Ai-Ho Liao, Chih-Hung Wang, Lin-Yi Chou, Yu-Chan Hung, Yi-Chun Lin, Ho-Chiao Chuang, Kuo-Hsing Ma, Hao-Li Liu, Jehng-Kang Wang, Cheng-Ping Shih

**Affiliations:** a Graduate Institute of Biomedical Engineering, National Taiwan University of Science and Technology, Taipei, Taiwan; b Department of Biomedical Engineering, National Defense Medical University, Taipei, Taiwan; c Department of Otolaryngology, Taipei City Hospital, Taipei, Taiwan; d Department of Otolaryngology–Head and Neck Surgery, Tri-Service General Hospital, National Defense Medical University, Taipei, Taiwan; e Graduate Institute of Medical Sciences, National Defense Medical University, Taipei, Taiwan; f Department of Mechanical Engineering, National Taipei University of Technology, Taipei, Taiwan; g Department of Biology and Anatomy, National Defense Medical University, Taipei, Taiwan; h Department of Electrical Engineering, National Taiwan University, Taipei, Taiwan; i Department of Biochemistry, National Defense Medical University, Taipei, Taiwan

**Keywords:** Blood–labyrinthine barrier, hearing loss, ischemia, metformin, oxygenated microbubbles, ultrasound, oxidative stress

## Abstract

Noise-induced hearing loss (NIHL) involves a biphasic pathophysiology. Intense noise exposure causes immediate cochlear vasoconstriction and ischemia, leading to transient hypoxia. Subsequent reperfusion triggers excess reactive oxygen species (ROS) production, resulting in oxidative stress and hair cell injury. This study therefore developed two oxygenated albumin microbubble (OMB) formulations—ionic-bond metformin-coated (iMet-OMBs) and covalent-bond metformin-encapsulated (cMet-OMBs)—and combined them with transcranial ultrasound (US) to enhance targeted delivery to the cochlea. This approach aims to provide transient oxygen supplementation while simultaneously reducing ROS-mediated injury. Microbubbles were characterized for morphology, oxygen loading, and metformin content. Based on their superior stability and drug-loading profile, cMet-OMBs were selected for *in vivo* evaluation. In a mouse NIHL model, animals were administered cMet-OMBs systemically via retro-orbital injection, followed by US triggering over the temporal bone. Auditory brainstem response (ABR) thresholds, cochlear oxygen tension, and outer hair cell (OHC) survival were assessed. US-mediated cMet-OMBs rupture transiently increased intracochlear oxygen tension, counteracting early hypoxia after noise exposure. Metformin released from cMet-OMBs attenuated ROS production through mitochondrial complex I inhibition and antioxidant pathway activation. Mice treated with cMet-OMBs + US showed significantly lower ABR threshold shifts and better OHC preservation compared with controls. This dual-action strategy combines transient oxygen supplementation from OMBs with sustained antioxidant protection from metformin. While oxygen delivery raises intracochlear oxygen tension, metformin suppresses ROS generation through mitochondrial complex I inhibition and AMPK/Nrf2 activation. This controlled, US-triggered release achieves net cochlear protection against NIHL without excessive oxidative burden.

## Graphical abstract

**Figure:**
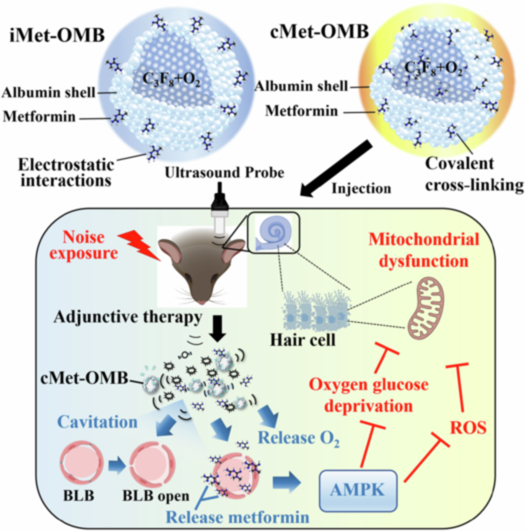

Metformin-coated (ionic bond) and metformin-encapsulated (covalent bond) oxygenated albumin microbubbles (iMet-OMBs and cMet-OMBs) were both prepared and characterized. In mice, cMet-OMBs were selected and injected into the blood. Transcranial ultrasound-mediated cavitation fragments cMet-OMBs within the cochlear microvasculature, transiently increasing BLB permeability and locally releasing oxygen. This targeted oxygen delivery, combined with metformin’s antioxidant properties, elevates intracochlear oxygen partial pressure while suppressing ROS, thereby protecting hair cells from noise-induced damage.

## Introduction

Noise-induced hearing loss (NIHL) is one of the most common inner ear disorders and shares pathological mechanisms with other cochlear injuries such as labyrinthine infarction, drug-induced ototoxicity, and presbyacusis, all of which can impair cochlear microcirculation and induce ischemic damage (Seidman et al. 1999). Experimental studies show that cochlear blood flow decreases within one hour after noise exposure (Lamm and Arnold 2000), and oxygen partial pressure in the perilymph declines during and after exposure. Subsequent reperfusion elicits a burst of reactive oxygen species (ROS) that overwhelms endogenous antioxidants, causing oxidative stress (Ohlemiller and Dugan 1999). These processes trigger apoptosis and inflammatory responses (Khan et al. 2010; Olivetto et al. 2015; Yang et al. 2021), resulting in hair cell and spiral ganglion neuron loss, stria vascularis damage, and elevated auditory brainstem response (ABR) thresholds (Taniguchi et al. 2002; Koga et al. 2003; Morizane et al. 2005; Lin et al. 2010). Inner hair cells are more vulnerable to ischemia than outer hair cells (Taniguchi et al. 2002; Mazurek et al. 2003), and the severity of cochlear damage depends on ischemic duration (Lin et al. 2010). Models of oxygen–glucose deprivation (OGD) further confirm that activation of Nrf2 and heme oxygenase−1 signaling can mitigate cochlear ischemic injury (Zhu et al. 2018; Zheng et al. 2023). Clinically, idiopathic sudden sensorineural hearing loss (ISSNHL) remains refractory in ~20% of treated patients (Chandrasekhar et al. 2019), and cochlear ischemia has been considered one of its etiologies (Chandrasekhar et al. 2019). Hyperbaric oxygen therapy is sometimes used for NIHL or ISSNHL, but the improvement in hearing recovery is limited (Buckey, 2019; Chandrasekhar et al. 2019).

Although several therapeutic strategies have been proposed, their effectiveness is insufficient. Hyperbaric oxygen increases oxygen supply but does not directly address ROS overproduction (Buckey, 2019; Chandrasekhar et al. 2019). Pharmacological antioxidants may reduce oxidative stress but show variable clinical benefits. Therefore, approaches that can both counteract hypoxia and suppress ROS generation are needed for effective NIHL therapy.

Metformin (N,N-dimethylbiguanide; MET) is a widely prescribed antidiabetic agent that also exerts protective effects against ischemia–reperfusion injury. In cardiac and neuronal models, MET activates AMP-activated protein kinase (AMPK), inhibits apoptotic signaling, restores mitochondrial function, and attenuates ROS production (Osorio-Llanes et al. 2023). Two population-based cohort studies reported that MET reduces the risk of ISSNHL and sensorineural hearing loss (Chen et al. 2019; Tseng, 2023). In addition, animal experiments demonstrated that MET protects against NIHL and cisplatin-induced ototoxicity (Kesici et al. 2018; Gedik et al. 2020; Liang et al. 2021; Kennedy et al. 2023). Together, these findings suggest that MET can provide otoprotection; however, prior studies relied on systemic administration, which is limited by the blood–labyrinth barrier (BLB) (Nyberg et al. 2019) and may lead to insufficient local drug concentrations in the cochlea.

Recent reviews have summarized the progress of US–microbubble (MB)–assisted inner ear drug delivery, highlighting its potential for enhancing cochlear permeability and therapeutic efficacy (Micaletti et al. 2023). However, these studies mainly focused on general MB-assisted delivery or other therapeutic agents, and none investigated the combined oxygen supplementation and antioxidant actions achieved in our work (Liao et al. 2016; Wen et al. 2021; Cesur et al. 2022). Our group previously demonstrated that ultrasound (US)-mediated MB cavitation can enhance drug delivery across the round window membrane (Shih et al. 2013). Other groups have shown that low-pressure pulsed US combined with MBs can transiently open the BLB and enable efficient therapeutic delivery into the cochlea (He et al. 2024). Nevertheless, no approach has yet achieved both improved drug delivery and increased intracochlear oxygenation simultaneously. To address this gap, we developed oxygenated albumin MBs loaded with MET, prepared in two formulations: ionic-bond coated (iMet-OMBs) and covalent-bond encapsulated (cMet-OMBs). We hypothesized that US-mediated cavitation of these MBs could deliver both oxygen and MET across the BLB, thereby alleviating hypoxia and oxidative stress in the cochlea. This dual-action strategy was evaluated in vitro using an OGD-induced cochlear ischemia model and *in vivo* in a mouse NIHL model, with treatments administered systemically via retro-orbital injection.

## Materials and methods

### MB synthesis and characterization

#### Preparation of metformin-encapsulated and metformin-coated oxygenated albumin microbubbles (cMet-OMBs and iMet-OMBs)

**Supplementary Figure 1**A shows that the preparations of cMet-OMBs were modified using existing methods; the resulting cMet-OMBs are held together by disulfide bonds between protein cysteine residues generated using ultrasonic emulsification method (Rahnama et al. 2015; Qin et al. 2024). The method used to prepare the cMet-OMBs was modified as follows: metformin (C_4_H_6_D_6_ClN_5_, molecular weight [MW] = 165.62, The United States Pharmacopeial Convention, Frederick, MD, USA) at concentrations of 177.32 μg/mL, 443.32 μg/mL, 709.28 μg/mL, and 886.6 μg/mL in 9.34 mL of phosphate-buffered saline (PBS) was mixed at 37 °C for 10 min, and 0.66 mL of 210 mg/mL HSA (Octapharma, Vienna, Austria) was then added, followed by incubation at 4 °C for 30 min. The sample was then subjected to 2 min of sonication with perfluorocarbon (C_3_F_8_) and oxygen (O_2_) gas using a sonicator (Branson Ultrasonics, Danbury, CT, USA). Only 6 mL of cMet-OMBs could be isolated after the sonication process. The sample was then centrifuged in 1 mL of PBS at 1,200 rpm (110 × g) for 2 min. The lower layer was removed to eliminate the free (unbound) metformin, 1 mL of PBS was added, and the sample was then stored at 4 °C.

**Supplementary Figure 1**B shows the self-assembly process used to produce iMet-OMBs. First, OMBs were prepared according to the procedure used in our previous studies (Liao et al. 2017; Liao et al. 2018; Liao et al. 2020a; Liao et al. 2021). Because albumin has negative charges, the surface potential of the albumin shell is less than zero; thus, it can attract molecules with positive charges. Under physiological conditions, MET has many amines (-NH_2_) that absorb hydrogen ions and transform into -NH_3_^+^ (positive charge). Therefore, an albumin shell with negative charges can be adsorbed onto MET by electrical adsorption. Various iMet-OMBs were prepared by incubating MET at concentrations of 82.81 μg/mL, 165.62 μg/mL, or 331.24 μg/mL with the produced OMBs on a rotary shaker (50 rpm; Shaker RS−01, TKS, New Taipei City, Taiwan) for 60 min at 4 °C in a refrigerator to produce iMet-OMBs. These iMet-OMBs were washed once to remove unbound MET. The amount of MET absorbed on the OMBs was measured at 233 nm using a Lambda 40 UV/VIS Spectrophotometer (PerkinElmer, Woodbridge, Canada) to calculate the adsorption efficiency of MET onto the OMBs, with the results substituted into the following equation:

(1) $$\mathrm{Adsorption}\;\mathrm{Efficiency}\;(\%)=\frac{\mathrm{Adsorption}\;\mathrm{capacity}\;(\mu g)\;}{\mathrm{Total}\;\mathrm{metformin}\;(\mu g)}\times 100\%$$

The size distributions of cMet-OMBs and iMet-OMBs in solution were measured using dynamic light scattering (SZ−100 Nanoparticle Analyzer, Horiba, Kyoto, Japan), and the numbers of cMet-OMBs and iMet-OMBs were measured using a MultiSizer III device (Beckman Colter, Fullerton, CA, USA) with a 30-μm aperture probe with a measurement range of 0.6–20 μm. The morphologies of the cMet-OMBs and iMet-OMBs were characterized by filtering the cMet-OMBs and iMet-OMBs using a 5-μm syringe filter (Sartorius, Goettingen, Germany), and 5 μL of the MB, OMB, cMet-OMBs and iMet-OMBs samples were then mounted on copper stubs with double-sided carbon adhesive tape and then coated with platinum (achieved at 0.1 nm/s and 30 mA for 60 seconds) using a JFC−1300 automatic sputter coater (JEOL, Tokyo, Japan). The morphologies of the MBs, OMBs, cMet-OMBs and iMet-OMBs were characterized using high-resolution field-emission scanning electron microscopy (FESEM; JSM-6500F, JEOL, Tokyo, Japan) at a 15 kV accelerating voltage.

### In vitro experiments

#### In vitro determination of the oxygen release kinetics of OMB after US triggering

For the in vitro release study, 3 mL of MB, OMB, or cMet-OMB suspension (at a concentration of 6−9.7 × 10^7^ bubbles/mL) was loaded into a dialysis bag (MW cutoff = 12,000–14,000), dialyzed against 100 mL of PBS release medium at pH 7.4 and 36.5–37.5 °C, and stirred using a magnetic bar at 600 rpm. The 1-MHz unfocused US transducer of the sonoporation system (plane-wave transducer module; Sonitron GTS, ST-TM1−12, Nepa Gene, Chiba, Japan) positioned 3 mm from the top of the dialysis bag under the liquid level provided sonication at a 3 W/cm^2^ power density for 30 seconds. The oxygen release kinetics were measured using an OxyLite 2000 with a bare fiber pO_2_ probe (Oxford Optronix, Oxford, United Kingdom). The OxyLite^TM^ fiber-optic oxygen sensor was placed on the outside of the dialysis bag 1 mm away and secured. All pO_2_ values were normalized to baseline values and expressed as changes in mmHg.

#### Optimization of US parameters for the transcranial destruction of cMet-OMBs

Because the enhancement of drug delivery is related to the destruction efficacy of MBs (Liao et al. 2020b), we investigated the US parameters required for cMet-OMB destruction in vitro by subjecting the MBs to US at 3 W/cm^2^ (*I*_SPTA_ = 672 mW/cm^2^; Sonitron GTS, Nepa Gene) for 1 min of US treatment. We set the US device (Nepa Gene) equipped with a 3 mm diameter probe to operate at a center frequency of 1 MHz and a duty cycle of 50%. The probe was placed directly onto the skull bone of each mouse using gel as a coupling agent. The skull bone was covered on a columnar chamber in the agarose phantom, which was filled with 1.5 mL of cMet-OMBs at 4 × 10^7^ MBs/mL. After US sonication, the cMet-OMBs solution in each well was imaged using a US animal imaging system (Prospect, S-Sharp Corporation, Taipei, Taiwan) to qualitatively visualize the presence and cavitation of MBs. Image analysis was used to estimate bubble destruction efficiency by comparing the signal intensity of cMet-OMBs before and after sonication. Importantly, US imaging was not used to measure oxygen or ROS; intracochlear oxygen partial pressure was measured separately with a OxyLite™ fiber-optic oxygen sensor, and ROS levels were assessed using DCFH-DA fluorescence assays as described below.

#### Cell culture, oxygen glucose deprivation model and pretreatment with iMet-OMB + US or cMet-OMB + US in vitro

The auditory HEI-OC1 cell line was kindly provided by Dr. Federico Kalinec (House Ear Institute, Los Angeles, CA, USA). Immunofluorescence staining for myosin 7a, a marker for cochlear hair cells, was performed, and myosin 7a expression was confirmed in the cells. HEI-OC1 cells were maintained under permissive conditions at 33 °C in high-glucose DMEM (Invitrogen, Waltham, MA, USA) supplemented with 10% fetal bovine serum and 5% CO₂, as described by Kalinec et al. (2016). Differentiation conditions were not applied in this study. Oxygen glucose deprivation conditions were established and utilized as an in vitro model of cochlear ischemia injury. The oxygen glucose deprivation model was applied to the cells in glucose-free DMEM and a N_2_/CO_2_ multigas incubator (APM-50D, Astec, Fukuoka, Japan) by setting 1% O_2_ low oxygen tension with a 94% N_2_ and 5% CO_2_ atmosphere at 33 °C for 16 h. Before exposure to oxygen glucose deprivation, MET, OMBs, iMet-OMBs and cMet-OMBs were added for 1 h. The treatments included groups of cells treated with or without US sonication. Twenty-four hours after oxygen glucose deprivation exposure, a cell viability assay was conducted by adding 500 µL of Alamar blue (1%) to each well for 1 h, and then the optical density (OD) of each culture well was measured with a microplate reader (Epoch^TM^) at excitation and emission wavelengths of 560 nm and 590 nm, respectively. The OD in the control cell group was taken to indicate a viability of 100%. For the TUNEL assay (Merck KGaA, Darmstadt, Germany), the cells were fixed in a 4% paraformaldehyde solution for 30 min, incubated with permeabilization solution for 2 min, and then incubated with TUNEL reaction mixture at 37 °C for 1 h. ROS levels were measured with the fluorescent dye Dichloro-dihydro-fluorescein diacetate (DCFH-DA) (D399; Thermo Fisher Scientific, Waltham, MA, USA). The cells were seeded in 24-well plates. After washing with PBS, medium containing 20 μM DCFH-DA was added to each well and incubated at 33 °C for 30 min. Dichlorofluorescein (DCF) fluorescence was measured using a fluorescence plate reader (Synergy H4 Hybrid Reader; BioTek Instruments, Winooski, VT) with excitation at 485 nm and emission at 528 nm.

### *In vivo* experiments

#### Noise exposure and animal treatment

The Institutional Animal Care and Use Committee of the National Defense Medical Center, Taipei, Taiwan, approved the experimental protocols (approval number: IACUC 23−137). A total of 37 male CBA/CaJ mice aged between 6 and 8 weeks and weighting 20−25 g, were used in this study. One mouse was sacrificed, and the skull bone was collected to evaluate the transcranial US parameters of cMet-OMB destruction. Thirty-six mice, including 15 mice for assessing oxygen partial pressure in the cochlea and 21 mice receiving noise exposure, were randomized into the no-treatment control, cMet-OMB and cMet-OMB + US groups (In the experiment of assessing oxygen partial pressure in the cochlea, *N* = 15, control group (*n* = 5), cMet-OMB group (*n* = 5), cMet-OMB + US group (*n* = 5); in the experiment of the animal model of noise exposure, *N* = 21, noise group (*n* = 7), cMet-OMB + noise group (*n* = 7), cMet-OMB + US + noise group (*n* = 7)). In this study, group sizes varied depending on the experimental design. For oxygen partial pressure measurements, invasive probe placement limited the feasible number of animals to *n* = 5 per group. For noise exposure and auditory brainstem response (ABR) experiments, larger cohorts (*n* = 7 per group) were used to ensure adequate statistical power. All sample sizes were determined based on our prior experience with similar models and were sufficient to achieve reliable statistical comparisons. In the manuscript, we use the common convention that capital *N* denotes the total number of animals in an experiment, whereas lowercase *n* indicates the number of animals per group.

The mice were anesthetized through the intraperitoneal injection of a mixture of xylazine (10 mg/kg; Rompun; Bayer, Leverkusen, Germany) and ketamine (40 mg/kg; Imalgene; Merial, Lyon, France). In the cMet-OMB group, cMet-OMBs were injected twice (50 μL, 4 × 10^7^ MBs/mL each time) via the retro-orbital injection technique, and the two injections were spaced 1 minute apart. In the cMet-OMB + US group, US sonoporation (*I*_SPTA_ = 672 mW/cm^2^, a duty cycle of 50%, twice, duration = 1 min each time) was applied simultaneously with each cMet-OMBs injection (twice, 50 μL, 4 × 10^7^ MBs/mL each time) at a position 3 mm away from the skull bone and corresponding to the tympanic bulla. In the noise exposure experiment, the cMet-OMB or cMet-OMB + US treatment was administered 1 h before noise exposure (cMet-OMB + noise and cMet-OMB + US + noise groups (*n* = 7 each)). The mice were subsequently placed in a soundproof booth with a loudspeaker (V12 HP, Tannoy, United Kingdom) mounted above the center of the cage, and both ears were exposed to white noise at a sound pressure level of 118 dB for 3 h. The sound intensity inside the chamber was tested using a sound level meter to ensure minimal deviations of sound intensity. One group receiving noise exposure without any treatment (serving as the control, noise group (*n* = 7)) simultaneously received 3 h of noise with the two treatment groups.

#### *In vivo* measurement of oxygen partial pressure in the cochlea

To verify that cMet-OMBs injected into mice combined with US can increase the degree of oxygen tension in the inner ear through BLB, the mice were anesthetized with xylazine and ketamine. After ensuring that the animals could breathe room air spontaneously, they were placed on an electric heating pad. After a postauricular incision was made, the soft tissues were bluntly dissected to expose the tympanic bulla. A 5-mm-diameter fenestration was created on the tympanic bulla to expose the cochlea and round window membrane under an operating microscope (F−170; Carl Zeiss, Germany). The round window membrane was punctured using a 29-gauge needle, and an OxyLite 2000 with a bare fiber pO2 probe (Oxford Optronix, Oxford, United Kingdom) was inserted into the scala tympani in the basal turn, after which the oxygen partial pressure of the perilymph in the cochlea was measured. The probe was inserted through the round window membrane without making any deliberate cuts. While penetration may cause slight, temporary disturbances in local oxygen levels, no intentional opening was created, and any resulting effect is considered negligible. After the oxygen partial pressure stabilized, OMBs and cMet-OMBs (50 μL, 4 × 10^8^ MBs/mL) were intravenously injected by using retro-orbital injection. Then, 3 W/cm^2^ (*I*_SPTA_ = 672 mW/cm^2^, duty cycle of 50%, twice, duration = 1 min each time) sonoporation was performed for 1 min, and the changes in the oxygen partial pressure were quantified by calculating the area under the curve. The mice (*N* = 15) were divided into 3 groups as follows: control group (*n* = 5), cMet-OMB group (*n* = 5), and cMet-OMB + US group (*n* = 5).

#### Auditory brainstem response recording

The hearing function of the mice was evaluated by measuring auditory brainstem responses (ABRs), as described previously (Lin et al. 2021). After the mouse was anesthetized and placed in the sound-attenuating chamber, subdermal needle electrodes were inserted at the vertex, behind the tested ear, and near the base of the tail. For the measurement of ABRs, specific stimuli (8, 16, and 24 kHz tone bursts) were produced using SigGen software (Tucker-Davis Technologies, Gainesville, FL, USA) and were then output monaurally to the external auditory canal using an insert earphone. The average responses from 1024 stimuli, at intensities ranging from 5–90 dB sound pressure level for each frequency, were acquired by lowering the sound intensity in 5 dB steps. The ABR threshold was defined as the lowest intensity at which a reproducible deflection in the evoked response trace could be identified. Threshold shift was defined as the difference in the threshold 2 weeks after noise exposure from before noise exposure. The mice (*N* = 21) were divided into 3 groups as follows: noise group (*n* = 7), cMet-OMB + noise group (*n* = 7), and cMet-OMB + US + noise group (*n* = 7).

#### Cochlear surface preparation

After ABRs were measured 2 weeks following noise exposure, the mice were euthanized (*N* = 21, noise group (*n* = 7), cMet-OMB + noise group (*n* = 7), cMet-OMB + US + noise group (*n* = 7)). Then the tympanic bulla was removed. The cochleae in the tympanic bulla were perfused and fixed with 4% paraformaldehyde for 1 h at room temperature and then decalcified. After washing with PBS, the bony capsule surrounding the cochlea, the cochlear lateral wall, and Reissner’s membrane were removed. The organ of Corti was carefully preserved. The samples were permeabilized with 0.3% Triton X−100 for 10 min. Then, the samples were incubated with an anti-myosin 7a antibody (Santa Cruz Biotechnology, Dallas, TX, USA) for 2 h, Alexa Fluor™ 647-conjugated phalloidin (1:500; Thermo Fisher Scientific, Waltham, MA, USA) for 30 min, and DAPI (4',6-diamidino−2-phenylindole; 1:1000; Thermo Fisher Scientific, Waltham, MA, USA) for 15 min. Images were obtained using an Zeiss LSM 880 confocal microscope (Carl Zeiss, Jena, Germany). The survival rates of outer hair cells (%) in the basal and middle turns of the cochlea were calculated using the following formula: survival rate (%) = 100 × [(the number of present outer hair cells/the number of present and lost outer hair cells in the organ of Corti)].

#### Statistical analysis

The data were analyzed statistically using the two-tailed Student’s t test to compare two groups. Multiple groups were compared using one-way ANOVA, followed by the Bonferroni multiple-comparisons test. A probability value of *p* < 0.05 was considered statistically significant.

## Results

### Characterization of cMet-OMBs and iMet-OMBs

**Figure 1.**
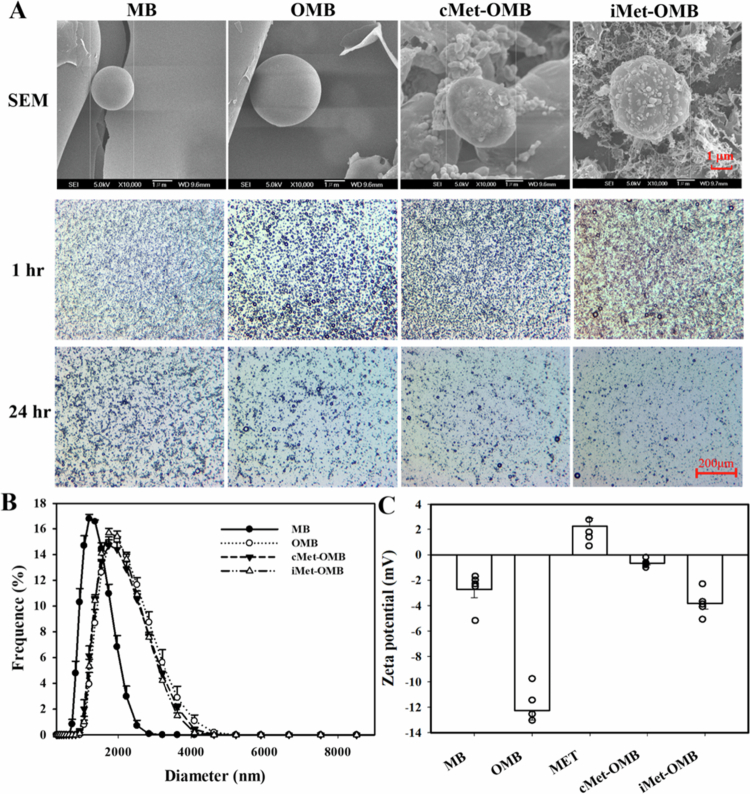
(A) SEM and brightfield microscope images of MBs, OMBs, cMet-OMBs and iMet-OMBs after standing for 1 and 24 h. (B) Frequency-dependent size distributions of MBs, OMBs, cMet-OMBs and iMet-OMBs and (C) zeta potentials of MBs, OMBs, MET, cMet-OMBs and iMet-OMBs. The values are expressed as the means ± SEMs (*n =* 5).

Figure 1A shows scanning electron microscopy (SEM) and brightfield microscopy images of MBs, OMBs, cMet-OMBs and iMet-OMBs after standing for 1 and 24 h. SEM images revealed the nanoscale complexes of cMet-OMBs and iMet-OMBs. The diameters of the MBs, OMBs, cMet-OMBs and iMet-OMBs were 1.25 ± 0.04 μm, 2.24 ± 0.14 μm, 1.89 ± 0.04 μm and 2.22 ± 0.25 μm, respectively (Figure 1B). The zeta potentials of MBs, OMBs, MET, cMet-OMBs and iMet-OMBs dispersed in aqueous solution were –2.74 ± 1.41 mV, –12.26 ± 1.70 mV, 2.2 4 ± 1.47 mV, –0.66 ± 0.30 mV and –3.82 ± 1.01 mV, respectively (Figure 1C). The changes in the zeta potentials of cMet-OMBs and iMet-OMBs (–0.66 ± 0.30 mV and –3.82 ± 1.01 mV) suggested the electrostatic interaction between the cargo and the carrier (Figure 1C). The concentrations of MBs, OMBs, cMet-OMBs and iMet-OMBs were 9.61 0.82 × 10^8^/mL, 6.67 ± 0.49 × 10^8^/mL, 9.56 ± 1.67 × 10^8^/mL and 2.8 ± 1.25 × 10^8^/mL, respectively.

To prepare the cMet-OMBs, the measured rates of MET encapsulation into OMBs were 24.92 ± 7.96% (1 mM original concentration of MET, 41.41 μg/mL), 45.35 ± 2.59% (2.5 mM original concentration of MET, 192.20 μg/mL), 65.50 ± 2.89% (4 mM original concentration of MET, 434.12 μg/mL), and 67.99 ± 1.51% (5 mM original concentration of MET, 570.23 μg/mL). To prepare the iMet-OMBs, the corresponding values of MET on the OMBs were 60.26 ± 8.53% (0.5 mM original concentration of MET, 49.90 μg/mL), 69.49 ± 2.98% (1 mM original concentration of MET, 115.09 μg/mL), and 40.10 ± 3.39% (2 mM original concentration of MET, 132.84 μg/mL). The encapsulation rates of cMet-OMBs for the 4 mM original concentration of MET were close to the saturation value, and the adsorption rates of iMet-OMBs for the 1 mM original concentration of MET were relatively stable during the experiments; therefore, we chose 4 mM for cMet-OMBs and 1 mM for iMet-OMBs as the amounts for all subsequent experiments.

### In vitro determination of the oxygen release kinetics of MBs, OMBs, and cMet-OMBs after US triggering

**Figure 2.**
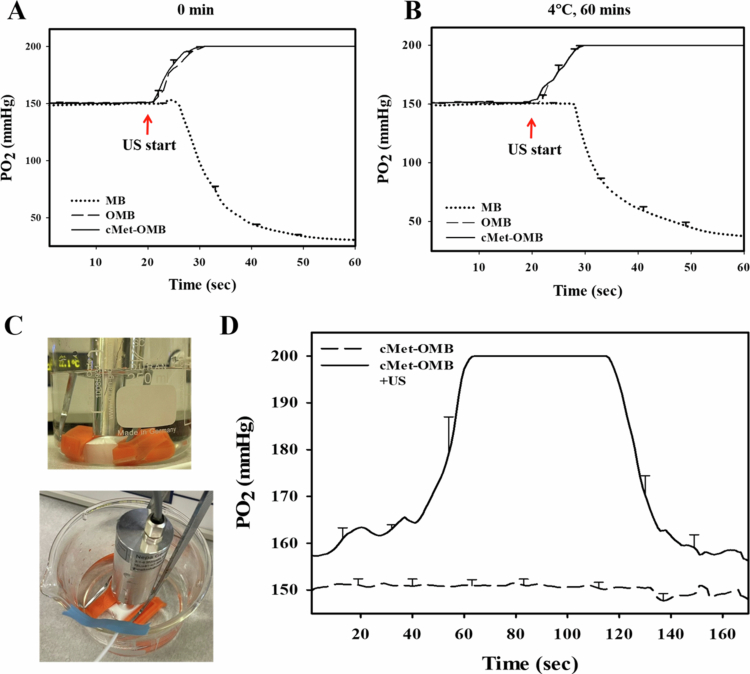
Oxygen release of MBs, OMBs and cMet-OMBs and US-mediated bubble destruction at pH 7.4. (A) Sixty-second oxygen release analysis of various MB solutions within 60 seconds after preparation. (B) Sixty-second oxygen release analysis of various MB solutions 1 h after preparation. (C) Schematic diagram of US-mediated in vitro oxygen release of MBs, OMBs, and cMet-OMBs in the dialysis bag. (D) One hundred eighty-second oxygen release analysis of cMet-OMBs with and without US using a dialysis bag. The oxygen sensor was placed on the outside of the dialysis bag 1 mm away (*n =* 5).

The experimental results in Figure 2A show that the oxygen partial pressures of OMBs and cMet-OMBs increased from 150 mmHg to 200 mmHg 60 seconds after the start of measurement, as OMBs and cMet-OMBs were encapsulated with certain proportions of oxygen. However, the oxygen partial pressure of MBs decreased from 150 mmHg to 20 mmHg after the start of US, as MBs do not contain oxygen. Moreover, as shown in Figure 2B, OMBs and cMet-OMBs prepared for 1 h could still reach an oxygen partial pressure of 200 mmHg. Figure 2C shows the ultrasonic probe acting on the MBs in the dialysis bag and the measurement of the partial pressure of oxygen in the surrounding environment of the dialysis bag. The 180-second oxygen partial pressure measurement results of cMet-OMBs with and without US (Figure 2D) revealed that when US was applied starting at 50 seconds, the oxygen partial pressure of the aqueous solution outside the dialysis bag increased to 200 mmHg 10 seconds after starting US irradiation and was maintained for approximately 60 seconds before returning to the initial oxygen partial pressure. This finding suggests that drug-loaded oxygen-containing MBs, including cMet-OMBs, can fully release and promote oxygen through the dialysis membrane to the outside environment by a US-induced cavitation effect.

ROS generation induced by US, OMB and the combination of OMB and US was investigated in HEI-OC1 cells (Supplementary Figure. 2). There was no significant difference in ROS levels among the control, US, OMB, and OMB + US groups. This finding suggests that OMB, US-induced OMB cavitation effect, and oxygen release from US-mediated OMBs do not increase ROS production.

#### Effects of iMet-OMB + US and cMet-OMB + US treatments on viability and ROS generation in HEI-OC1 cells under oxygen glucose deprivation conditions

**Figure 3.**
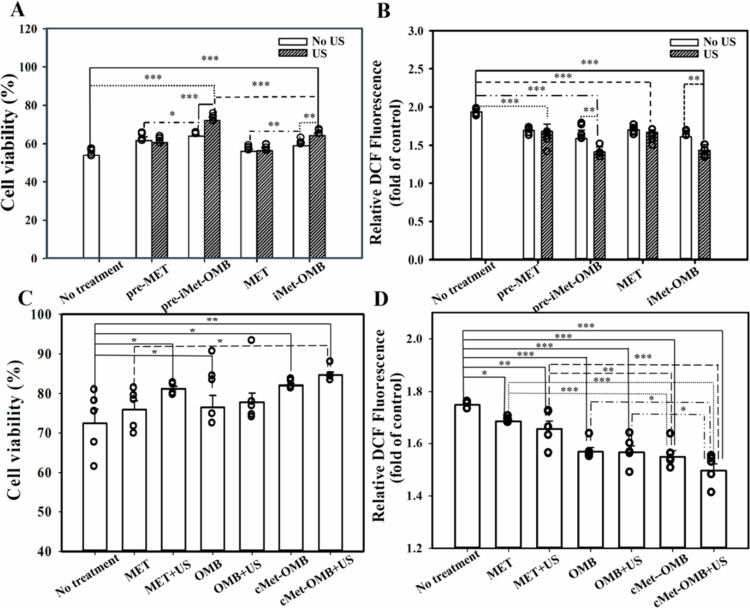
(A) Viability of HEI-OC1 cells after oxygen glucose deprivation exposure only (no treatment), pretreatment with MET with or without US followed by oxygen glucose deprivation exposure (pre-MET), pretreatment with iMet-OMBs with or without US followed by oxygen glucose deprivation exposure (pre-iMet-OMBs), MET with or without US during oxygen glucose deprivation exposure (MET), and iMet-OMBs with or without US during oxygen glucose deprivation exposure (iMet-OMB). Cell viability was expressed as a percentage relative to the controls without oxygen glucose deprivation exposure. (B) Comparison of ROS levels between the groups receiving the same conditions as in (A). The ROS levels of the groups were assessed with a DCFH-DA staining and are expressed as the value relative to that of the controls without oxygen glucose deprivation exposure. (C) Cell viability of HEI-OC1 cells after oxygen glucose deprivation exposure only (no treatment), pretreatment with MET without US followed by oxygen glucose deprivation exposure (MET), pretreatment with MET with US followed by oxygen glucose deprivation exposure (MET + US), pretreatment with OMBs without US followed by oxygen glucose deprivation exposure (OMB), pretreatment with OMBs with US followed by oxygen glucose deprivation exposure (OMB + US), pretreatment with cMet-OMBs without US followed by oxygen glucose deprivation exposure (cMet-OMB), and pretreatment with cMet-OMBs with US followed by oxygen glucose deprivation exposure (cMet-OMB + US). The cell viability was expressed as a percentage relative to the controls without oxygen glucose deprivation exposure. (D) Comparison of ROS levels between the groups receiving the same conditions as in (C). The ROS levels of the groups are expressed as the values relative to those of the controls without oxygen glucose deprivation exposure. The data are expressed as the means ± SEMs, with *n =* 5 for each bar. *p < 0.05, **p < 0.01, ***p < 0.001.

In the iMet-OMBs experiment, the results in Figure 3A show that oxygen glucose deprivation exposure without treatment led to a decrease in the viability of HEI-OC1 cells to 53.93 ± 0.84%. Pretreatment with MET or iMet-OMBs alone did not improve oxygen glucose deprivation-induced cell death. However, pretreatment with iMet-OMBs with US significantly increased cell viability to 71.97 ± 0.82% (pre-iMet-OMB + US group vs. no treatment group, *p* < 0.001). The TUNEL assay revealed the most TUNEL-positive cells in the no-treatment group and the fewest TUNEL-positive cells in the pre-iMet-OMB + US group (Supplementary Figure 3). There was a significant difference in the number of TUNEL-positive cells between the two groups. These findings suggest that pretreatment with iMet-OMBs with US ameliorates oxygen glucose deprivation-induced apoptosis in HEI-OC1 cells. Different treatments were administered during oxygen glucose deprivation exposure, and the therapeutic effects on the viability of HEI-OC1 cells were compared. Compared with the control group, the iMet-OMB + US treatment group presented greater cell viability (64.31 ± 0.5% vs. 53.93 ± 0.84%, *p* < 0.001). The effect of iMet-OMB + US on oxygen glucose deprivation-induced oxidative stress was evaluated (Figure 3B). The ROS levels in the pre-iMet-OMB + US and iMet-OMBs + US groups were significantly lower than that in the no-treatment group. These results suggest that administration of iMet-OMBs with US can decrease oxygen glucose deprivation-induced damage and ROS generation in auditory cells. The effects of pretreatment with cMet-OMBs and US on the viability and ROS generation in HEI-OC1 cells exposed to oxygen glucose deprivation were subsequently evaluated. Figure 3C shows that the cell viability in the cMet-OMB + US group was the highest among all the groups and was significantly greater than that in the control group (84.63 ± 0.8% vs. 72.45 ± 3.6%, *p* = 0.004). As shown in Figure 3D, the ROS level in the cMet-OMB + US group was the lowest among all the groups and was significantly lower than that in the control group (*p* < 0.001). These findings revealed that cMet-OMB + US can attenuate oxygen glucose deprivation-induced death and oxidative stress in auditory cells. Because covalent bonds on MBs are more stable than noncovalent bonds for carrying drugs during the trafficking of MBs through blood vessels, cMet-OMBs were selected and used for the subsequent animal studies.

### Optimization of sonication parameters for the transcranial enhancement of inner ear drug delivery using high-frequency US imaging

**Figure 4.**
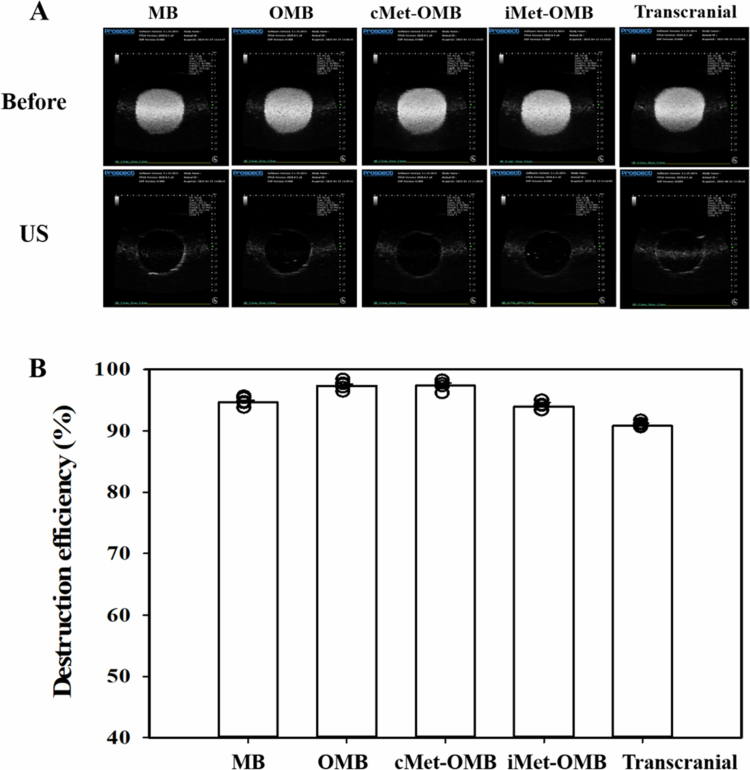
High-frequency US images of MBs, OMBs, cMet-OMBs, and iMet-OMBs without and with US sonication. (A) Representative images of MBs, OMBs, cMet-OMBs and iMet-OMBs before and after 1 min of US sonication. ‘Transcranial’ indicates cMet-OMBs with 1 min of sonication through the skull bones of the mice. (B) Quantification of MB destruction at the same concentration with 1 min of sonication. The data are expressed as the means ± SEMs, with *n =* 5 for each bar.

To perform transcranial US for the enhancement of inner ear drug delivery, a high-frequency US imaging system was used to evaluate the optimal sonication parameters. One-minute US sonication at a power density of 3 W/cm^2^ (*I*_SPTA_ = 672 mW/cm^2^; Sonitron GTS, Nepa Gene) was applied to different types of MBs (Figure 4). Without the skull bones of the mice, the destruction efficiencies of MBs, OMBs, cMet-OMBs and iMet-OMBs were 94.63 ± 0.71%, 97.28 ± 0.77%, 97.36 ± 0.90%, and 94.00 ± 0.57%, respectively. The destruction efficiency of cMet-OMBs following 1 min of sonication through the skull bone was 90.85 ± 0.21%. Therefore, this US parameter can be used for transcranial US-mediated MB cavitation and was deemed the optimal setting for conducting subsequent *in vivo* experiments.

### Effect of transcranial US-mediated cMet-OMBs on oxygen partial pressure in the cochlea and NIHL in mice

**Figure 5.**
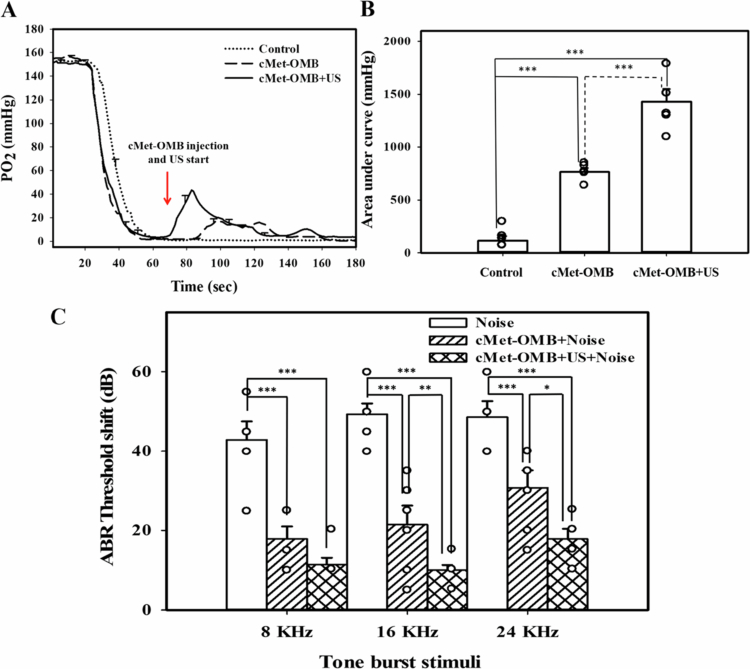
(A) *In vivo* dynamic oxygen partial pressure analysis of the cochlea. Perilymph of the basal turn was measured in the three groups, including the control (no treatment), cMet-OMB (only cMet-OMB injection and no US), and cMet-OMB + US (transcranial US-mediated cMet-OMB) groups. The arrow indicates the start of cMet-OMB injection in the cMet-OMB group and the start of transcranial US-mediated cMet-OMB injection in the cMet-OMB + US group. (B) Integrated area under curve analysis of the results in (A). The results are expressed as the mean ± SEM, with *n =* 5 for each bar. ***p < 0.001. (C) The ABR threshold shifted 2 weeks after noise exposure in the Noise, cMet-OMB + Noise, and cMet-OMB + US + Noise groups. The results are expressed as the mean ± SEM, with *n* = 7 for each bar. *p < 0.05, **p < 0.01, ***p < 0.001.

**Figure 6.**
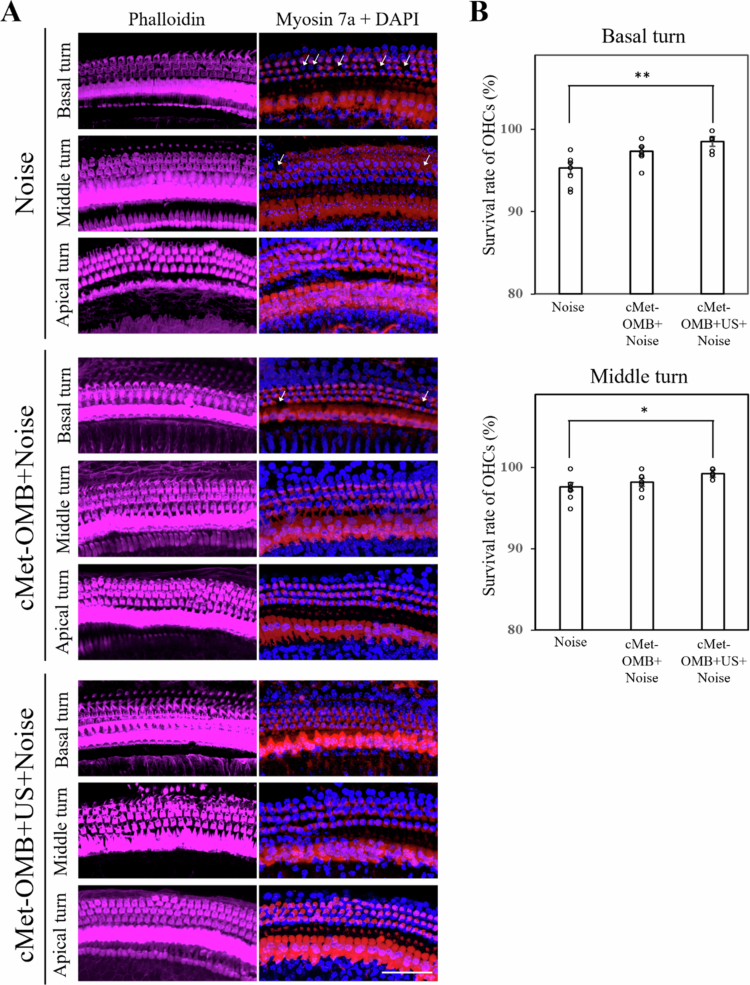
(A) Representative images of surface preparations of the basal, middle and apical turns of the cochleae from the three groups on Day 14 after noise exposure. Immunofluorescence staining shows filamentous actin (violet, phalloidin), nuclei (blue, DAPI) and cochlear hair cells (red, myosin 7a). The arrows indicate the loss of outer hair cells (OHCs). (B) Comparison of the survival rates of OHCs in the basal and middle turns of cochleae from each group. The results are expressed as the mean ± SEM, with *n* = 7 for each bar. Scale bars = 50 μm. *p < 0.05, **p < 0.01.

As shown in Figure 5A, the oxygen partial pressure in the cochlea of the control group was maintained at 1 mmHg. In the cMet-OMBs group, the cochlear oxygen partial pressure reached 20 mmHg 20 seconds after the start of cMet-OMBs injection (at the 90th second) and slowly decreased to 1 mmHg 60 seconds postinjection (at the 130th second). The cochlear oxygen partial pressure in the cMet-OMB + US group rapidly increased after the start of transcranial administration of US-mediated cMet-OMBs and reached 50 mmHg. It slowly decreased to 1 mmHg at the 160th second. As shown in Figure 5B, the areas under the curve from the 70th to the 180th second in the control, cMet-OMB, and cMet-OMB + US groups were 117.80 ± 96.10, 764.40 ± 85.20, and 1429.64 ± 270.35 mmHg, respectively. The area under the curve in the cMet-OMB + US group was significantly greater than those in the other two groups (cMet-OMB + US vs. the other two groups, *p* < 0.001). These results suggest that transcranial US-mediated cMet-OMBs can effectively increase oxygen tension in the cochlea. The preventative effect of transcranial US-mediated cMet-OMBs on NIHL was subsequently evaluated. The hearing function results in Figure 5C show significant differences in the threshold shift at 8, 16 and 24 kHz stimuli among the three groups. The threshold shifts in the cMet-OMB + Noise and cMet-OMB + US + Noise groups were lower than those in the Noise group. Compared with the cMet-OMB + Noise group, the cMet-OMB + US + Noise group presented lower threshold shifts with 16 and 24 kHz stimuli. Figure 6 shows the severity of outer hair cell loss in cochleae from different treatment groups compared on Day 14 after noise exposure. No outer hair cell loss was observed in the apical turn of the cochlea in any of the three groups. In the Noise group, the survival rates of outer hair cells in the basal and middle turns of the cochlea were 95.26 ± 0.67% and 97.63 ± 0.48%, respectively. In the cMet-OMB + Noise group, the survival rates of outer hair cells in the basal and middle turns were 97.30 ± 0.51% and 98.17 ± 0.47%, respectively. In the cMet-OMB + US + Noise group, the survival rates of outer hair cells in the basal and middle turns were 98.50 ± 0.59% and 99.25 ± 0.20%, respectively. The survival rates of outer hair cells of the middle and basal turns in the cMet-OMB + US + Noise group were greater than those in the Noise group (basal turn, *p* = 0.008; middle turn, *p* = 0.036). There was no significant difference in the outer hair cell survival rates between the cMet-OMB + Noise and Noise groups (basal turn, *p* = 0.199; middle turn, *p* = 1). As shown in Figure 5 and 6, the results revealed that cMet-OMBs injection may have some protective effect on NIHL. The combination of transcranial US irradiation and cMet-OMBs can result in better protection against NIHL than treatment with cMet-OMBs alone. Taken together, these findings demonstrated that transcranial US-mediated cMet-OMBs improved intracochlear oxygen tension and attenuated noise-induced cochlear damage in mice.

## Discussion

Compared with prior MET-based otoprotection studies that relied on systemic administration (Kesici et al. 2018; Chen et al. 2019; Nyberg et al. 2019; Gedik et al. 2020; Liang et al. 2021; Kennedy et al. 2023; Micaletti et al. 2023; Tseng, 2023), our approach directly targets the cochlea and combines MET delivery with an oxygen pulse. This dual-action strategy simultaneously addresses acute hypoxia and subsequent oxidative stress, thereby overcoming the limitations of systemic dosing, which is restricted by the BLB and may result in insufficient cochlear concentrations. Similarly, previous US–MB approaches to the inner ear have largely focused on enhancing round window membrane or BLB permeability to facilitate drug or nanoparticle entry, such as through transcanal or transcranial sonication, tight-junction modulation, or low-pressure pulsed US (Shih et al. 2013; Kalinec et al. 2016; Liao et al. 2021; Micaletti et al. 2023). While effective for delivery, these strategies lack oxygen supplementation and therefore cannot directly mitigate ischemia-related injuries.

By contrast, oxygen micro/nanobubbles in oncology have been used to transiently reoxygenate hypoxic tissue, but phospholipid-shelled formulations typically circulate briefly and rupture prematurely, limiting their therapeutic window (Yang et al. 2018; Reusser et al. 2020; Tan et al. 2021). In our study, albumin-shelled, oxygenated Met-OMBs offered greater stability and enabled US-triggered release across the intact skull, producing an immediate but localized rise in intracochlear pO₂ consistent with OMB release kinetics. Importantly, the concomitant delivery of MET provided sustained suppression of ROS via mitochondrial complex I inhibition and AMPK/Nrf2 activation. Together, these complementary actions resulted in superior ABR threshold preservation and enhanced survival of outer hair cells, underscoring the novelty and therapeutic potential of our dual-mechanism platform compared to approaches that emphasize either delivery or oxygenation alone.

In our present study, a high-efficiency manufacturing method was used to prepare drug-loaded OMBs with high concentration and high internal stability in the blood to develop a novel treatment for inner ear diseases. OMBs can be efficiently loaded with drugs and oxygen and then release drugs and oxygen precisely via US. Ionic bond-coated MET and covalent bond-encapsulated MET OMBs (iMet-OMBs and cMet-OMBs) were both prepared and demonstrated to have protective and therapeutic effects on oxygen glucose deprivation-induced auditory damage and NIHL. The SEM images revealed that the surface of cMet-OMBs were smoother than those of iMet-OMBs. Covalent and noncovalent interactions are the two main forces that hold atoms together. Relatively strong covalent bonds arise from sharing electrons, but ionic noncovalent interactions involve weaker forces (Guo et al. 2020). Therefore, cMet-OMBs, which hold MET and albumin together by covalent disulfide bonds between protein cysteine residues, were selected, injected, and allowed to enter the inner ear through the vasculature in *in vivo* experiments.

In the in vitro experiment, MET alone did not increase the viability of auditory cells following oxygen glucose deprivation exposure. The administration of iMet-OMBs or cMet-OMBs with US irradiation significantly improved oxygen glucose deprivation-induced cell death. The effects of iMet-OMBs or cMet-OMBs administration combined with US on oxygen glucose deprivation-induced damage were greater than those of iMet-OMBs or cMet-OMBs without US. Because US can trigger iMet-OMB and cMet-OMB destruction to efficiently deliver oxygen and MET to auditory cells, the best protective effects of iMet-OMBs and cMet-OMBs can be achieved following US irradiation. Moreover, treatment with MET alone slightly diminished oxygen glucose deprivation-induced ROS generation in auditory cells. The administration of iMet-OMBs or cMet-OMBs with US led to a greater reduction in oxygen glucose deprivation-induced ROS generation. These findings suggest that US-mediated cMet-OMBs or cMet-OMBs can effectively attenuate ischemic damage and oxidative stress in the cochlea. Some studies have reported that excess oxygen supplementation may induce oxidative stress and an inflammatory response (Gerstner et al. 2008; Pannu, 2016). In this study, we demonstrated that oxygen delivery via US-mediated OMBs does not increase ROS production. Furthermore, the OMB and OMB + US groups were pretreated with oxygen glucose deprivation-exposed auditory cells to evaluate their effects on ROS generation and cell survival. The administration of OMBs or OMBs with US did not lead to an increase in oxidative stress or the deterioration of oxygen glucose deprivation-induced cell death. The results showed that OMB administration with US in this study was a safe application method for auditory cells. Previous studies have demonstrated that local oxygen delivery by different techniques has beneficial effects on ischemic models (Swyer et al. 2014; Guan et al. 2021; He et al. 2023; Ho et al. 2023; Zhong et al. 2023). Codelivery of therapeutic agents and oxygen significantly enhances the therapeutic effect of these agents on ischemic lesions (Guan et al. 2021; Zhong et al. 2023). Oxygenated MB cavitation improves oxygenation of the targeted area, modulates endothelial metabolism, and inhibits hypoxic, apoptotic and inflammatory factors (Ho et al. 2023). US-mediated oxygenated MBs prevent ischemia‒reperfusion injury in murine hindlimbs and hearts (Ho et al. 2023). Oxygen-carrying nanoparticles alleviate hypoxic tissue damage and ameliorate brain and myocardial infarction in mice (Swyer et al. 2014; He et al. 2023). Oxygen-releasing microspheres were co-delivered with mesenchymal stem cells to the ischemic limb, and better tissue regeneration and stem cell survival were reported (Zhong et al. 2023). The combined effects of stem cell-derived exosomes and oxygen-carrying nanoparticles synergistically have been found to promote angiogenesis and muscle regeneration (Guan et al. 2021). The findings of this study were similar to those of previous studies (Guan et al. 2021; Zhong et al. 2023). US-induced cavitation of iMet-OMBs or cMet-OMBs had a greater beneficial effect on oxygen glucose deprivation-induced damage and oxidative stress than did MET alone. These findings suggest that the administration of drugs supplemented with oxygen resulted in better treatment outcomes for auditory ischemia injury.

In experiments on the destruction efficiency of cMet-OMBs induced by ultrasonic waves on mouse skull bone, this parallel pulsed ultrasonic wave with an acoustic intensity of ISPTA = 672 mW/cm^2^ was able to fully break MBs after 1 min of application and was subsequently applied for the *in vivo* study. In the group with transcranial US (the cMet-OMB + US group), the oxygen partial pressure level of the cochlea quickly increased to 50 mmHg, whereas in the group without US (the cMet-OMB group), the oxygen partial pressure level slightly increased to 20 mmHg within a period of time after cMet-OMB injection. The quantification of the area under the oxygen partial pressure curve in the cMet-OMB + US group was the highest among the three groups. These findings suggest that the setting of transcranial US-mediated OMBs in this study can facilitate BLB permeability and oxygen release in the inner ear. Systemic injection of cMet-OMBs without US is unlikely to elevate intracochlear oxygen tension substantially, as MBs cannot cross the BLB. Any modest increase would mainly reflect systemic oxygen release into circulation. By contrast, US-mediated cavitation fragments the MBs within the cochlear microvasculature, transiently increasing BLB permeability and locally releasing oxygen, thereby producing the targeted rise in intracochlear pO₂ observed in our study. Therefore, a prominent increase in cochlear oxygen tension was detected in the mice that received transcranial US-mediated cMet-OMBs. Furthermore, transcranial US-mediated cMet-OMBs were applied for the prevention of NIHL. The hearing and morphological results revealed that transcranial US-mediated cMet-OMBs had better protection against NIHL than cMet-OMBs alone. These findings demonstrate that transcranial US-mediated cMet-OMBs represent an effective prevention modality for NIHL and could be applied to treat inner ear diseases in the future.

MET has been demonstrated to trigger mitophagy in auditory hair cells (HEI-OC1 line) via activation of the AMPK pathway, thereby protecting against apoptosis in oxidative-stress models (Muri et al. 2019). In animal models of pneumococcal meningitis, MET preserved spiral ganglion neurons and improved auditory thresholds, suggesting it may have protective effects in the inner ear (Chang et al. 2014). MET is a highly hydrophilic, positively charged molecule. Its entry into cells generally depends on organic cation transporters and related membrane-bound carriers (Lai et al. 2025). However, there are no studies to identify which specific transporters or receptors mediate MET uptake in cochlear tissues *in vivo*. In this study, US-mediated cavitation delivers both oxygen and MET across the BLB, alleviating acute hypoxia and activating MET’s AMPK antioxidant pathways. This process reduces ROS and protects cochlear hair cells. The observed auditory protection evidenced by ABR threshold preservation and the survival of outer hair cells indirectly supports cochlear delivery. Pharmacokinetic profiling of metformin in cochlear fluids/tissues will be an important aim for future research.

## Conclusion

This study first investigated the therapeutic and preventative effects of MET coated with OMBs combined with US in an ex vivo model of auditory ischemia injury and NIHL *in vivo*. MET-loaded OMBs with US irradiation effectively attenuated ischemic damage and oxidative stress in auditory cells. The partial pressure levels of oxygen in the in vitro oxygen release model showed that cMet-OMBs can fully and stably release oxygen to the target area. We further demonstrated that transcranial US-mediated cMet-OMBs effectively increased oxygen partial pressure in the cochlea. In the NIHL model, the application of transcranial US-mediated cMet-OMBs improved hearing and outer hair cell loss. In conclusion, transcranial US-mediated cMet-OMBs have potential for treating ischemia-associated inner ear diseases, including ISSNHL.

## Supplementary Material

Supplementary Material

## Data Availability

The data presented in this study are available on request from the corresponding author.
