# Supplementary material for: Transcranial ultrasound combined with intravenous metformin-loaded oxygenated microbubbles attenuates noise-induced hearing loss in mice
Source: Drug Deliv. 2025 Nov 5;32(1):2576220. doi: 10.1080/10717544.2025.2576220 (PMC12599344; doi:10.1080/10717544.2025.2576220)
Supplement: Supplementary Material [file IDRD_A_2576220_SM0880.docx]

Transcranial ultrasound combined with intravenous metformin-loaded oxygenated microbubbles attenuates noise-induced hearing loss in mice

Ai-Ho Liao^1,2,#^, Chih-Hung Wang^3,4,5,#^, Lin-Yi Chou^1^, Yu-Chan Hung^1^, Yi-Chun Lin^4^, Ho-Chiao Chuang^6^, Kuo-Hsing Ma^7^, Hao-Li Liu^8^, Jehng-Kang Wang^9^, and Cheng-Ping Shih^4,^*

1. Graduate Institute of Biomedical Engineering, National Taiwan University of Science and Technology, Taipei 106335, Taiwan.
2. Department of Biomedical Engineering, National Defense Medical University, Taipei 114201, Taiwan.
3. Department of Otolaryngology, Taipei City Hospital, Taipei 103212, Taiwan.
4. Department of Otolaryngology–Head and Neck Surgery, Tri-Service General Hospital, National Defense Medical University, Taipei 114202, Taiwan.
5. Graduate Institute of Medical Sciences, National Defense Medical University, Taipei 114201, Taiwan.
6. Department of Mechanical Engineering, National Taipei University of Technology, Taipei 106344, Taiwan.
7. Department of Biology and Anatomy, National Defense Medical University, Taipei 114201, Taiwan.
8. **Department of Electrical Engineering, National Taiwan University,** Taipei 106319, Taiwan.
9. Department of Biochemistry, National Defense Medical University, Taipei 114201, Taiwan.

# Contributed equally:

Name: Ai-Ho Liao and Chih-Hung Wang

*Corresponding author

Cheng-Ping Shih, M.D., Ph.D.

Department of Otolaryngology–Head and Neck Surgery, Tri-Service General Hospital, National Defense Medical University, Taipei 11490, Taiwan.


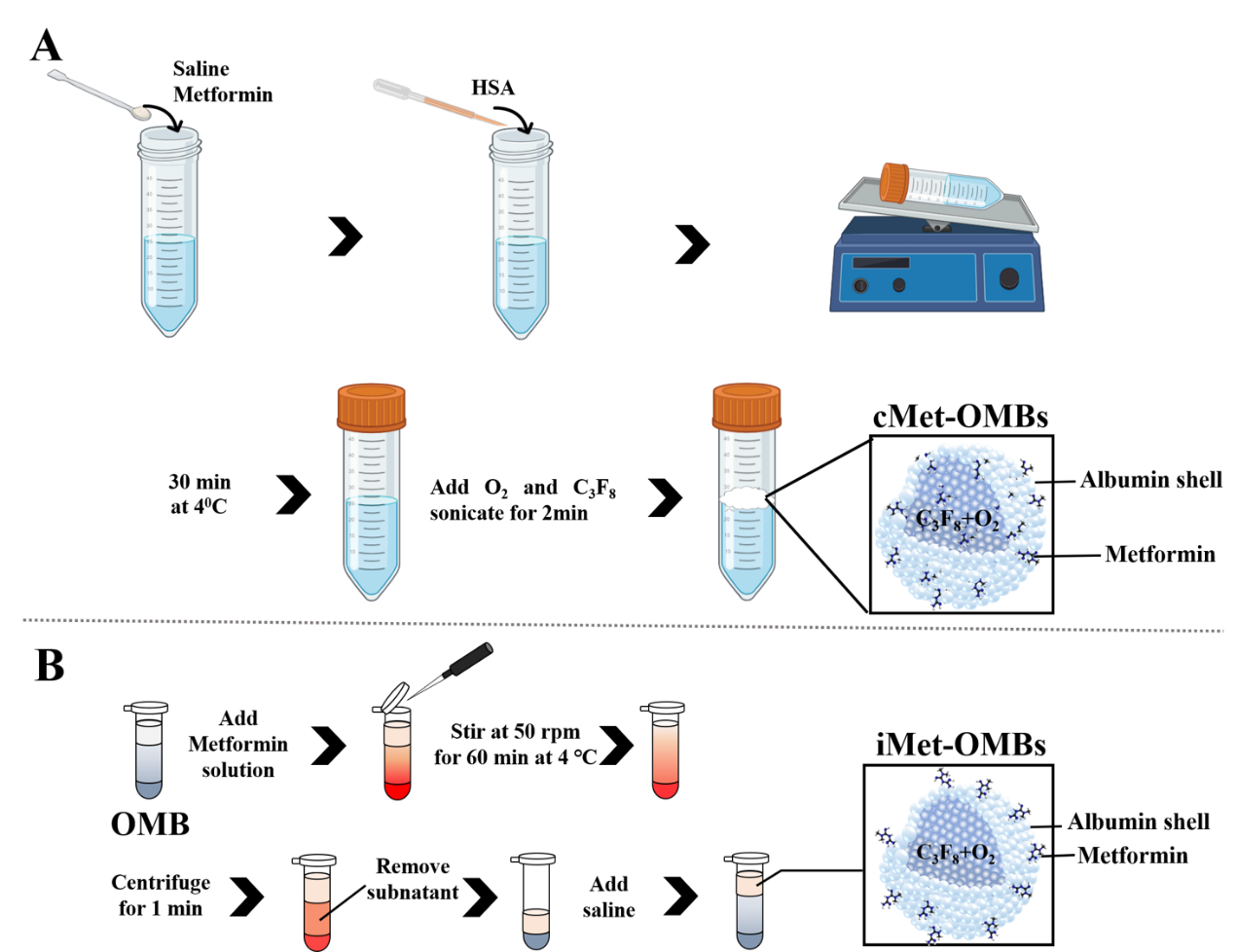


**Supplementary Fig. 1**. Schematic (not to scale) of the MET (A) encapsulating and (B) self-assembly processes used to produce cMet-OMBs and iMet-OMBs.


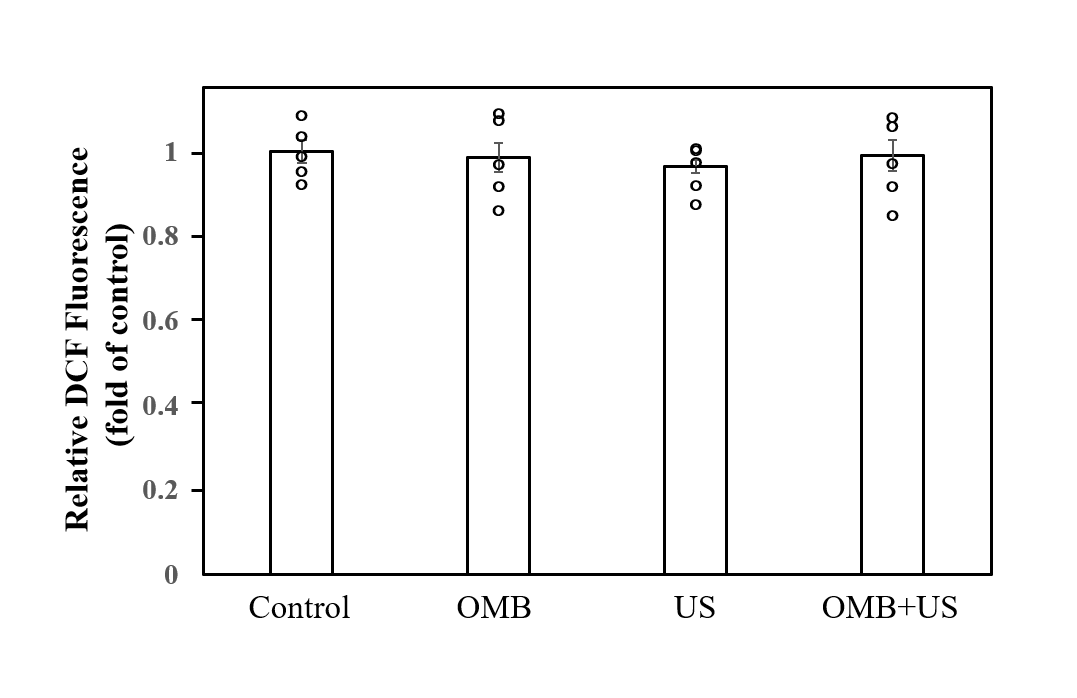


**Supplementary Fig. 2.** Comparison of ROS levels in HEI-OC1 cells under different conditions: untreated control, treatment with OMBs (OMB), exposure to ultrasound (US), and combined treatment with OMBs and ultrasound (OMB+US). Data are presented as means ± SEM, with n=5 for each group.


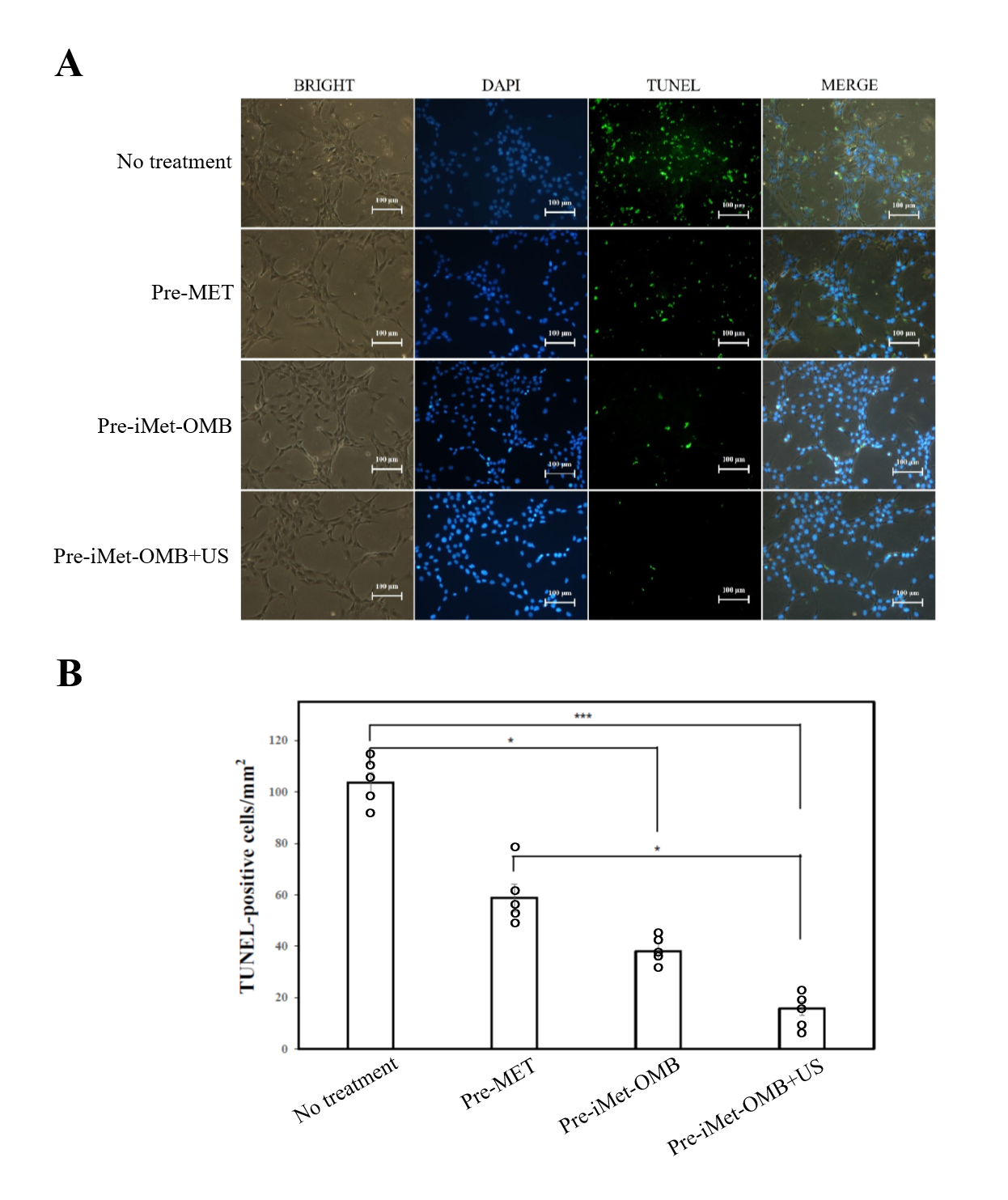


**Supplementary Fig. 3**. Pretreatment with iMet-OMBs with US ameliorated oxygen glucose deprivation-induced apoptosis in HEI-OC1 cells. (A) Representative image of the TUNEL assay and (B) comparison of TUNEL-positive cells between the groups, including no treatment (oxygen glucose deprivation exposure without treatment), pre-MET (pretreatment with MET without US followed by oxygen glucose deprivation exposure), Pre-iMet-OMB (pretreatment with iMet-OMBs without US followed by oxygen glucose deprivation exposure), and Pre-iMet-OMB+US (pretreatment with iMet-OMBs with US followed by oxygen glucose deprivation exposure) groups. The data are expressed as the means ± SEMs, with *n=*5 for each bar. **p* < 0.05, ****p* < 0.001.
